# Supplementary material for: Abolishing storage lipids induces protein misfolding and stress responses in Yarrowia lipolytica
Source: J Ind Microbiol Biotechnol. 2023 Sep 23;50(1):kuad031. doi: 10.1093/jimb/kuad031 (PMC10563384; doi:10.1093/jimb/kuad031)
Supplement: kuad031_Supplemental_File [file kuad031_supplemental_file.docx]

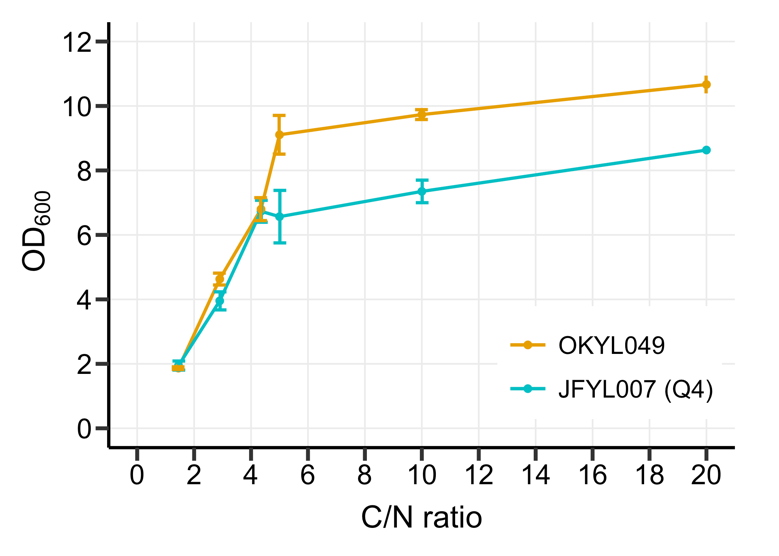


**Figure S1**: *Y. lipolytica* was grown in delft media for 72 hours. The media composition was kept constant, but the glucose concentration was varied to produce C/N ratios between 1.45 and 20. The OD_600_ was measured and plotted against the C/N ratio. We tested the Q4 strain (JFYL007) and an obese strain (OKYL049) as they show opposite phenotypes that might affect the transition between carbon or nitrogen limitation. The obese strain may experience carbon limitation for higher C/N ratios than the wild-type due to lipid overproduction. Q4 lacks the genes responsible for TAGs synthesis. OKYL049 (*DGA1* overexpression and *are1* deletion) accumulates high levels of TAGs. C/N ratios between 1.45 and 4.43 are carbon limiting for both strains. At higher C/N ratios, the nitrogen becomes limiting, and increasing the glucose concentration doesn’t have a major effect on the final OD_600_. Dots represent the average OD_600_ of triplicates, and error bars represent the standard deviation.


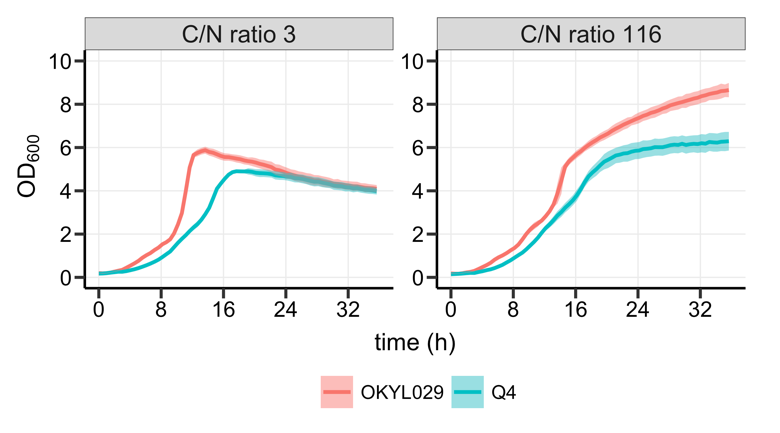


**Figure S2**: Strains OKYL029 and Q4 were cultured in 96-wells plates with C/N ratio 3 (C-lim, left panels) or C/N ratio 116 (N-lim, right panels). OD_600_ was measured with the growth profiler every 30 minutes. The curves represent the average of triplicates, and the shadowed areas represent the standard deviation.


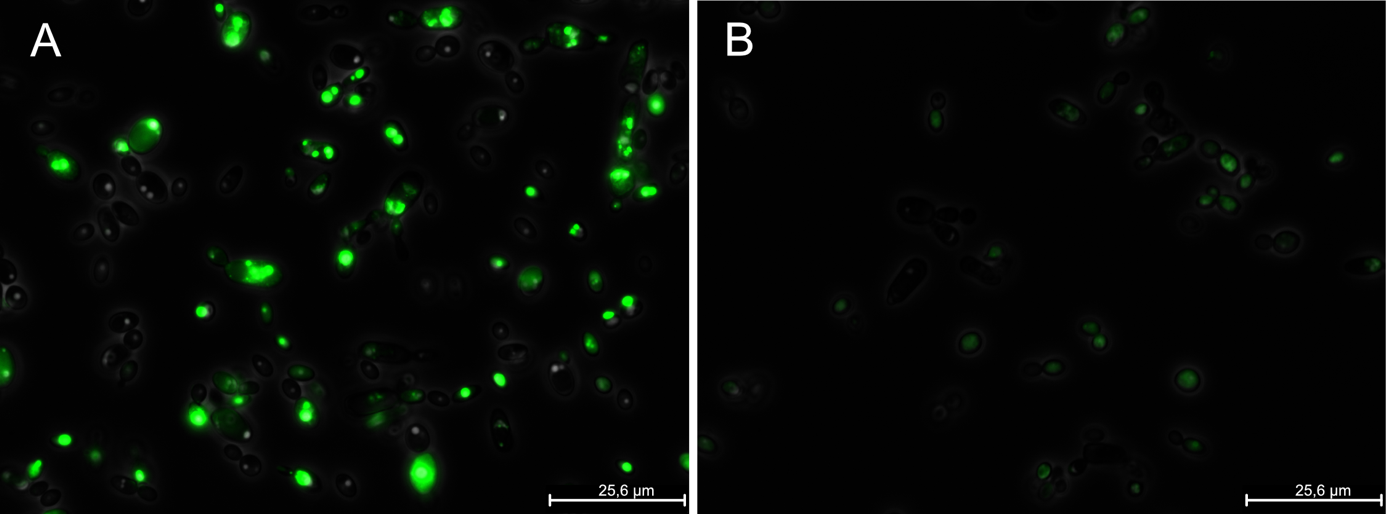


**Figure S3:** Microscope pictures of *Y. lipolytica* strain OKYL029 (A) and Q4 (B) grown under N-lim. Cells were cultured in N-lim Novogy media (0.5 g/L urea, 1.5 g/L yeast extract, 0.85 g/L casamino acids, 1.7 g/L yeast nitrogen base (YNB) without amino acids and ammonium sulphate, 5.1 g/L potassium hydrogen phthalate, 100.0 g/L glucose, pH set to 5.5 with KOH) for 72 hours and stained with Bodipy® Lipid Probe. In the wild-type strain OKYL029 (A) lipid droplets (LD) are visible. No visible lipid droplets (LD) were observed in the Q4 strain.


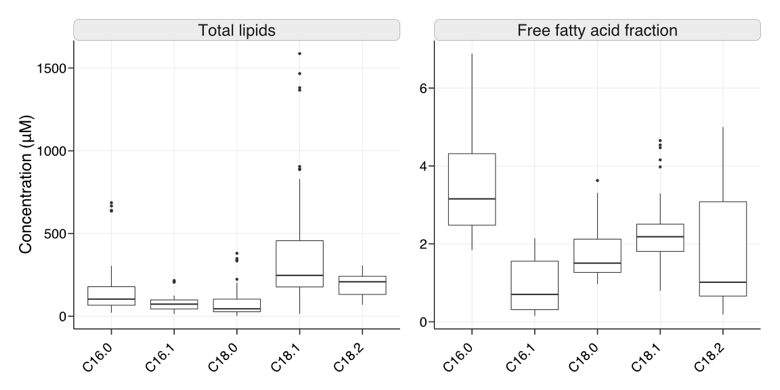


**Figure S4:** Molar concentrations of each fatty acid (C16:0, C16:1, C18:0, C18:1, C18:2) both in total lipids and in the FFA fraction. The concentrations of lipids have been calculated based on the FAME and SPE data of strains OKYL029 and Q4 grown on C-lim (C/N ratio 3) and N-lim (C/N ratio 116). The highest concentration measured is 1500 µM for the total lipids, while for the FFA fraction is 6 μM.

**Table S1:** Differentially expressed genes and their protein functions. Differentially expressed genes were extracted comparing the Q4 strain to OKYL029 on C/N ratio 3. The adjusted p-value cut-off is 0.05 and the absolute log2 fold change cut-off is 0,5. Protein functions were retrieved from UniProt (05.05.2022).

| genes | Protein.names | P.Value | adj.P.Val | logFC |
| --- | --- | --- | --- | --- |
| YALI1_A21372g | Triacylglycerol lipase (EC 3.1.1.3) | 4,94E-04 | 3,45E-02 | -1,30E+00 |
| YALI1_B12451g | Proteasome complex subunit Rpn13 ubiquitin receptor-domain-containing protein | 1,25E-04 | 1,06E-02 | -4,55E-01 |
| YALI1_D27995g | Enhancer of translation termination 1 | 4,40E-04 | 3,15E-02 | 1,11E+00 |
| YALI1_E01694g | Sensitive to high expression protein 9, mitochondrial | 2,02E-06 | 4,15E-04 | -1,15E+00 |
| YALI1_E38859g | Glycerophosphocholine phosphodiesterase (EC 3.1.4.2) | 2,06E-07 | 6,33E-05 | 2,85E+00 |
| YALI1_F33246g | Glycolipid 2-alpha-mannosyltransferase-domain-containing protein | 2,99E-05 | 3,17E-03 | -1,32E+00 |

**Table S2:** Differentially expressed genes and their protein functions. Differentially expressed genes were extracted comparing the Q4 strain to OKYL029 on C/N ratio 116. The adjusted p-value cut-off is 0.05 and the absolute log2 fold change cut-off is 0,5. Protein functions were retrieved from UniProt (05.05.2022).

| genes | Protein.names | P.Value | adj.P.Val | logFC |
| --- | --- | --- | --- | --- |
| YALI1_A00375g | Tyrosine-protein phosphatase domain-containing protein | 4,90E-05 | 5,25E-04 | 2,18E+00 |
| YALI1_A00801g | Alpha-N-methyltransferase NTM1 | 1,99E-05 | 2,62E-04 | 1,85E+00 |
| YALI1_A01173g | Serine/threonine-protein phosphatase (EC 3.1.3.16) | 4,17E-03 | 1,75E-02 | 9,41E-01 |
| YALI1_A01298g | Endoplasmic reticulum-based factor for assembly of V-ATPase-domain-containing protein | 1,72E-10 | 2,21E-08 | -3,10E+00 |
| YALI1_A01394g | Cation_ATPase_N domain-containing protein | 4,65E-03 | 1,91E-02 | -5,34E-01 |
| YALI1_A01618g | SRCR domain-containing protein | 5,32E-04 | 3,68E-03 | -1,13E+00 |
| YALI1_A02083g | Prefoldin subunit 4 | 3,16E-05 | 3,78E-04 | -1,66E+00 |
| YALI1_A02263g | FYVE-type domain-containing protein | 1,08E-02 | 3,66E-02 | -6,12E-01 |
| YALI1_A02394g | Peptidase A1 domain-containing protein | 1,50E-02 | 4,64E-02 | 4,57E-01 |
| YALI1_A02682g | MULE domain-containing protein | 8,63E-03 | 3,11E-02 | 1,13E+00 |
| YALI1_A03170g | S-adenosyl-L-methionine-dependent methyltransferase | 3,20E-03 | 1,45E-02 | -1,85E+00 |
| YALI1_A04119g | Ribosome production factor 2 homolog (Ribosome biogenesis protein RPF2 homolog) | 7,61E-03 | 2,82E-02 | 1,31E+00 |
| YALI1_A04893g | Spindle pole body component | 5,78E-04 | 3,90E-03 | -2,08E+00 |
| YALI1_A06453g | Very-long-chain 3-oxoacyl-CoA reductase (EC 1.1.1.330) (3-ketoacyl-CoA reductase) (3-ketoreductase) (KAR) (Microsomal beta-keto-reductase) | 2,01E-09 | 1,87E-07 | 2,36E+00 |
| YALI1_A07712g | Deoxyhypusine hydroxylase (DOHH) (EC 1.14.99.29) (Deoxyhypusine dioxygenase) (Deoxyhypusine monooxygenase) | 1,47E-03 | 7,98E-03 | 2,73E+00 |
| YALI1_A08856g | Peptidylprolyl isomerase (EC 5.2.1.8) | 4,55E-03 | 1,88E-02 | -6,21E-01 |
| YALI1_A09638g | PRA1 family protein | 1,69E-09 | 1,63E-07 | 2,84E+00 |
| YALI1_A09856g | Mitochondrial intermediate peptidase | 9,54E-03 | 3,36E-02 | -7,95E-01 |
| YALI1_A10934g | E3 ubiquitin ligase complex SCF subunit | 1,34E-02 | 4,26E-02 | 1,09E+00 |
| YALI1_A12411g | Brix domain-containing protein | 5,67E-04 | 3,85E-03 | 1,66E+00 |
| YALI1_A13349g | Cysteine protease (EC 3.4.22.-) | 1,49E-02 | 4,61E-02 | 5,16E-01 |
| YALI1_A14390g | Histone acetyltransferases subunit 3-domain-containing protein | 1,72E-05 | 2,36E-04 | -1,07E+00 |
| YALI1_A15073g | Ribonuclease H (RNase H) (EC 3.1.26.4) | 9,78E-03 | 3,41E-02 | -7,56E-01 |
| YALI1_A15846g | Kinetochore protein SPC25 | 8,73E-04 | 5,40E-03 | 8,47E-01 |
| YALI1_A16028g | RNase H type-1 domain-containing protein | 3,49E-03 | 1,54E-02 | -4,59E-01 |
| YALI1_A16178g | SWIM-type domain-containing protein | 1,65E-02 | 4,97E-02 | 8,67E-01 |
| YALI1_A17927g | SMK-1 domain-containing protein | 1,85E-03 | 9,50E-03 | -1,52E+00 |
| YALI1_A18557g | 40S ribosomal protein S0 | 1,08E-02 | 3,66E-02 | 1,67E+00 |
| YALI1_A18817g | SAGA-associated factor 11 | 9,82E-04 | 5,80E-03 | 1,35E+00 |
| YALI1_A18847g | Acyl-protein thioesterase 1 | 9,47E-04 | 5,66E-03 | 1,17E+00 |
| YALI1_A19862g | MFS domain-containing protein | 1,62E-02 | 4,90E-02 | 1,08E+00 |
| YALI1_A20149g | PIH1 domain-containing protein | 1,06E-03 | 6,18E-03 | 1,34E+00 |
| YALI1_A20398g | Aldedh domain-containing protein | 1,92E-04 | 1,61E-03 | 1,04E+00 |
| YALI1_A21372g | Triacylglycerol lipase (EC 3.1.1.3) | 8,91E-03 | 3,18E-02 | -1,55E+00 |
| YALI1_A21409g | HECT-type E3 ubiquitin transferase (EC 2.3.2.26) | 6,67E-03 | 2,53E-02 | -1,09E+00 |
| YALI1_A21797g | 116 kDa U5 small nuclear ribonucleoprotein component (U5 snRNP-specific protein, 116 kDa) | 3,47E-04 | 2,61E-03 | -1,30E+00 |
| YALI1_A22157g | NUC153 domain-containing protein | 3,86E-05 | 4,40E-04 | 1,07E+00 |
| YALI1_A22454g | TauD domain-containing protein | 1,71E-06 | 4,05E-05 | -1,81E+00 |
| YALI1_B00578g | Methylenetetrahydrofolate reductase (EC 1.5.1.20) | 5,41E-03 | 2,15E-02 | -9,11E-01 |
| YALI1_B01325g | N-alpha-acetyltransferase 40 (EC 2.3.1.257) | 1,61E-03 | 8,53E-03 | -6,27E-01 |
| YALI1_B01628g | Nucleolar protein 58 | 9,65E-03 | 3,37E-02 | 8,07E-01 |
| YALI1_B02091g | Transcription elongation factor Spt6 | 1,49E-05 | 2,13E-04 | -1,55E+00 |
| YALI1_B02345g | 3-isopropylmalate dehydratase (EC 4.2.1.33) (Alpha-IPM isomerase) (Isopropylmalate isomerase) | 9,78E-04 | 5,80E-03 | 1,32E+00 |
| YALI1_B02375g | Peptidase family M41-domain-containing protein | 7,06E-03 | 2,65E-02 | -1,01E+00 |
| YALI1_B02733g | Lysophospholipase (EC 3.1.1.5) | 3,69E-04 | 2,71E-03 | -5,77E-01 |
| YALI1_B04068g | E1-E2 ATPase-domain-containing protein | 2,69E-04 | 2,11E-03 | 9,85E-01 |
| YALI1_B04228g | Peptidase_S8 domain-containing protein | 9,47E-03 | 3,33E-02 | -4,98E-01 |
| YALI1_B04950g | MT-A70-domain-containing protein | 4,13E-03 | 1,74E-02 | -1,57E+00 |
| YALI1_B05454g | Vacuolar protein sorting-associated protein 27 | 1,39E-03 | 7,73E-03 | -1,15E+00 |
| YALI1_B05483g | Putative molybdopterin binding domain-domain-containing protein | 4,72E-03 | 1,93E-02 | 1,22E+00 |
| YALI1_B05522g | P-loop containing nucleoside triphosphate hydrolase protein | 6,51E-04 | 4,25E-03 | 9,32E-01 |
| YALI1_B05646g | 60S ribosomal protein L42 | 5,91E-03 | 2,28E-02 | 1,87E+00 |
| YALI1_B06726g | Yos1-like protein | 1,14E-02 | 3,80E-02 | 5,71E-01 |
| YALI1_B07598g | Non-specific serine/threonine protein kinase (EC 2.7.11.1) | 3,99E-04 | 2,87E-03 | -6,90E-01 |
| YALI1_B08123g | Pantoate--beta-alanine ligase (EC 6.3.2.1) (Pantoate-activating enzyme) (Pantothenate synthetase) | 9,02E-04 | 5,48E-03 | 3,10E+00 |
| YALI1_B08898g | Zn(2)-C6 fungal-type domain-containing protein | 1,94E-03 | 9,83E-03 | 1,04E+00 |
| YALI1_B09211g | Aldo_ket_red domain-containing protein | 6,02E-06 | 1,08E-04 | 2,76E+00 |
| YALI1_B09310g | Long chronological lifespan protein 2 | 3,67E-03 | 1,60E-02 | 1,32E+00 |
| YALI1_B09837g | Tr-type G domain-containing protein | 7,73E-06 | 1,29E-04 | -1,35E+00 |
| YALI1_B10032g | PH domain-containing protein | 1,72E-07 | 7,07E-06 | 1,09E+00 |
| YALI1_B11794g | Bms1-type G domain-containing protein | 9,00E-03 | 3,20E-02 | 1,28E+00 |
| YALI1_B11853g | Hsp70 protein-domain-containing protein | 2,45E-03 | 1,18E-02 | 2,24E+00 |
| YALI1_B12451g | Proteasome complex subunit Rpn13 ubiquitin receptor-domain-containing protein | 1,61E-02 | 4,89E-02 | -7,01E-01 |
| YALI1_B13662g | HotDog domain-containing protein | 1,51E-02 | 4,66E-02 | 1,57E+00 |
| YALI1_B13723g | PKS_ER domain-containing protein | 9,23E-03 | 3,27E-02 | 1,74E+00 |
| YALI1_B16158g | Metallophos domain-containing protein | 4,23E-03 | 1,77E-02 | 1,18E+00 |
| YALI1_B16584g | Enhancer of polycomb-like protein | 1,02E-02 | 3,53E-02 | -1,78E+00 |
| YALI1_B17039g | DNA-(apurinic or apyrimidinic site) lyase (EC 4.2.99.18) | 2,42E-03 | 1,17E-02 | 2,77E+00 |
| YALI1_B17441g | Ferric reductase like transmembrane component-domain-containing protein | 1,15E-03 | 6,63E-03 | -1,07E+00 |
| YALI1_B18368g | RRM domain-containing protein | 7,55E-03 | 2,81E-02 | -2,57E+00 |
| YALI1_B18887g | Fn3_like domain-containing protein | 8,08E-03 | 2,96E-02 | -3,46E-01 |
| YALI1_B19120g | Double-strand break repair protein | 1,14E-02 | 3,81E-02 | -1,53E+00 |
| YALI1_B19217g | Carbamoyl-phosphate synthase L chain, ATP binding domain-domain-containing protein | 5,47E-04 | 3,74E-03 | 1,43E+00 |
| YALI1_B19520g | Histone-lysine N-methyltransferase, H3 lysine-4 specific (EC 2.1.1.354) | 1,52E-02 | 4,67E-02 | 7,97E-01 |
| YALI1_B19774g | HAD_SAK_1 domain-containing protein | 3,52E-04 | 2,63E-03 | -1,76E+00 |
| YALI1_B20130g | SVP1-like protein 2 | 7,91E-05 | 7,80E-04 | 3,50E+00 |
| YALI1_B20870g | Kinase-like domain-containing protein | 1,17E-02 | 3,83E-02 | -1,06E+00 |
| YALI1_B21538g | Amino acid permease/ SLC12A domain-containing protein | 2,99E-03 | 1,38E-02 | -2,03E+00 |
| YALI1_B22266g | Ubiquitin-like domain-containing protein | 9,29E-09 | 6,50E-07 | 1,94E+00 |
| YALI1_B22793g | Calponin homology domain-containing protein | 5,56E-07 | 1,66E-05 | -2,81E+00 |
| YALI1_B22908g | Ribosomal protein L17 | 4,07E-07 | 1,32E-05 | 2,46E+00 |
| YALI1_B23017g | Major facilitator superfamily domain-containing protein | 1,66E-05 | 2,29E-04 | 1,69E+00 |
| YALI1_B23420g | RNA exonuclease 4 | 9,59E-03 | 3,36E-02 | 1,12E+00 |
| YALI1_B23794g | Queuine tRNA-ribosyltransferase accessory subunit 2 (Queuine tRNA-ribosyltransferase domain-containing protein 1) | 2,73E-06 | 6,04E-05 | 1,75E+00 |
| YALI1_B24672g | DDE-1 domain-containing protein | 5,73E-03 | 2,23E-02 | 1,39E+00 |
| YALI1_B25952g | F-box domain-containing protein | 5,18E-04 | 3,61E-03 | 1,78E+00 |
| YALI1_B26430g | CNH domain-domain-containing protein | 2,80E-04 | 2,19E-03 | -1,10E+00 |
| YALI1_B27254g | Mitochondrial import inner membrane translocase subunit TIM50 | 1,42E-02 | 4,46E-02 | 1,16E+00 |
| YALI1_B27500g | Fe2OG dioxygenase domain-containing protein | 2,43E-03 | 1,17E-02 | 3,16E+00 |
| YALI1_B27874g | D-fructose-6-phosphate amidotransferase (EC 2.6.1.16) (Hexosephosphate aminotransferase) | 5,53E-09 | 4,47E-07 | -2,77E+00 |
| YALI1_B27968g | TauD domain-containing protein | 1,91E-03 | 9,70E-03 | -1,57E+00 |
| YALI1_B28098g | Frag1/DRAM/Sfk1 family-domain-containing protein | 1,09E-02 | 3,68E-02 | -1,70E+00 |
| YALI1_B29266g | BTB domain-containing protein | 1,23E-04 | 1,13E-03 | -1,91E+00 |
| YALI1_B29505g | ATP-dependent RNA helicase DBP6 | 2,02E-03 | 1,01E-02 | 1,55E+00 |
| YALI1_B29915g | Peptidase_S8 domain-containing protein | 9,99E-05 | 9,40E-04 | -6,09E-01 |
| YALI1_C00258g | Carboxylic ester hydrolase (EC 3.1.1.-) | 8,57E-04 | 5,36E-03 | 1,23E+00 |
| YALI1_C00503g | 5'-AMP-activated protein kinase beta subunit, interation domain-domain-containing protein | 1,15E-03 | 6,65E-03 | 2,28E+00 |
| YALI1_C01789g | SNF2 family N-terminal domain-domain-containing protein | 8,39E-03 | 3,05E-02 | 7,74E-01 |
| YALI1_C01876g | RPAP3_C domain-containing protein | 8,87E-04 | 5,46E-03 | -3,31E+00 |
| YALI1_C02188g | Exocyst complex component Sec10 | 4,71E-03 | 1,93E-02 | 7,89E-01 |
| YALI1_C02215g | Phosphatidylinositol 4-kinase (EC 2.7.1.67) | 5,98E-04 | 3,98E-03 | 2,40E+00 |
| YALI1_C02550g | Methylmalonate-semialdehyde dehydrogenase (CoA acylating) (EC 1.2.1.27) | 3,67E-04 | 2,70E-03 | 3,67E+00 |
| YALI1_C04436g | WD40-repeat-containing domain protein | 6,79E-05 | 6,81E-04 | 1,32E+00 |
| YALI1_C04490g | Phosphatase 2C-like domain-containing protein | 1,16E-02 | 3,83E-02 | -9,01E-01 |
| YALI1_C04570g | HSP20-like chaperone | 4,39E-03 | 1,81E-02 | 1,19E+00 |
| YALI1_C05931g | MARVEL domain-containing protein | 1,12E-02 | 3,77E-02 | 1,46E+00 |
| YALI1_C06394g | Putative gamma-glutamylcyclotransferase | 5,08E-03 | 2,05E-02 | 3,07E+00 |
| YALI1_C06681g | Aminotran_1_2 domain-containing protein | 9,49E-06 | 1,52E-04 | 1,61E+00 |
| YALI1_C07028g | CRAL-TRIO domain-containing protein | 6,40E-03 | 2,45E-02 | 3,22E+00 |
| YALI1_C08242g | Vacuolar fusion protein MON1 | 4,06E-08 | 2,27E-06 | -1,74E+00 |
| YALI1_C09851g | Apurinic-apyrimidinic endonuclease 2 (DNA-(apurinic or apyrimidinic site) endonuclease 2) | 1,94E-05 | 2,61E-04 | -1,51E+00 |
| YALI1_C09885g | Homeobox domain-containing protein | 3,22E-03 | 1,45E-02 | -2,17E+00 |
| YALI1_C10534g | BTB domain-containing protein | 1,77E-03 | 9,17E-03 | 1,71E+00 |
| YALI1_C11441g | Big_8 domain-containing protein | 1,12E-04 | 1,04E-03 | 1,26E+00 |
| YALI1_C12220g | MFS domain-containing protein | 1,20E-02 | 3,91E-02 | 1,00E+00 |
| YALI1_C12619g | NADP-dependent oxidoreductase domain-containing protein | 6,19E-09 | 4,76E-07 | 3,18E+00 |
| YALI1_C13007g | Cytochrome c oxidase assembly protein COX20, mitochondrial | 2,08E-03 | 1,03E-02 | -6,66E-01 |
| YALI1_C13560g | Spindle pole body component | 8,92E-04 | 5,46E-03 | 8,75E-01 |
| YALI1_C13768g | AA_permease domain-containing protein | 1,66E-03 | 8,74E-03 | 6,00E-01 |
| YALI1_C13817g | Altered inheritance of mitochondria protein 21 | 6,73E-06 | 1,16E-04 | -1,78E+00 |
| YALI1_C13890g | Peptidyl-prolyl cis-trans isomerase (PPIase) (EC 5.2.1.8) | 1,79E-03 | 9,26E-03 | -1,75E+00 |
| YALI1_C15297g | Thymidylate synthase (EC 2.1.1.45) | 8,63E-06 | 1,42E-04 | 2,66E+00 |
| YALI1_C15526g | 2-Hacid_dh_C domain-containing protein | 1,36E-05 | 2,00E-04 | 1,29E+00 |
| YALI1_C16294g | Cytochrome b5 heme-binding domain-containing protein | 5,68E-03 | 2,22E-02 | 1,14E+00 |
| YALI1_C16851g | Membrane-associating domain-domain-containing protein | 5,21E-03 | 2,10E-02 | -1,14E+00 |
| YALI1_C18396g | BHLH domain-containing protein | 3,31E-08 | 1,88E-06 | 1,28E+00 |
| YALI1_C18541g | Nudix hydrolase domain-containing protein | 2,01E-04 | 1,66E-03 | -1,20E+00 |
| YALI1_C18932t | CCHC-type domain-containing protein | 8,36E-05 | 8,14E-04 | 2,50E+00 |
| YALI1_C18948t | RNA-directed DNA polymerase (EC 2.7.7.49) | 8,98E-09 | 6,43E-07 | 2,84E+00 |
| YALI1_C19840g | Transcriptional regulator of RNA polII, SAGA, subunit-domain-containing protein | 4,01E-03 | 1,70E-02 | -1,34E+00 |
| YALI1_C22922g | 60S ribosomal protein L17 | 8,90E-03 | 3,18E-02 | 1,18E+00 |
| YALI1_C23359g | DH domain-containing protein | 7,67E-03 | 2,84E-02 | 1,22E+00 |
| YALI1_C23526g | Altered inheritance of mitochondria protein 31, mitochondrial | 1,46E-04 | 1,29E-03 | 1,70E+00 |
| YALI1_C23548g | Mitochondrial ribosomal protein L28-domain-containing protein | 1,57E-02 | 4,80E-02 | 1,20E+00 |
| YALI1_C24081g | BZIP domain-containing protein | 6,66E-11 | 1,21E-08 | 2,44E+00 |
| YALI1_C24434g | Poly [ADP-ribose] polymerase (PARP) (EC 2.4.2.-) | 2,85E-12 | 1,46E-09 | 3,68E+00 |
| YALI1_C25307g | eIF-2B GDP-GTP exchange factor subunit alpha | 5,36E-05 | 5,63E-04 | 1,52E+00 |
| YALI1_C25352g | 2-methoxy-6-polyprenyl-1,4-benzoquinol methylase, mitochondrial (EC 2.1.1.201) (Ubiquinone biosynthesis methyltransferase COQ5) | 4,16E-05 | 4,64E-04 | 1,69E+00 |
| YALI1_C25512g | Amine oxidase (EC 1.4.3.-) | 5,96E-12 | 2,29E-09 | 5,33E+00 |
| YALI1_C25598g | Tryptophanyl-tRNA synthetase (EC 6.1.1.2) | 5,15E-03 | 2,08E-02 | -2,08E+00 |
| YALI1_C26533g | RING-type domain-containing protein | 5,40E-04 | 3,72E-03 | 7,60E-01 |
| YALI1_C28497g | Methylthioribulose-1-phosphate dehydratase (MTRu-1-P dehydratase) (EC 4.2.1.109) | 1,42E-05 | 2,05E-04 | -2,44E+00 |
| YALI1_C28664g | Peptidase S8/S53 domain-containing protein | 3,31E-03 | 1,48E-02 | -6,45E-01 |
| YALI1_C29506g | Nitric oxide dioxygenase (EC 1.14.12.17) | 1,71E-04 | 1,47E-03 | -2,83E+00 |
| YALI1_C29538g | MFS domain-containing protein | 4,08E-04 | 2,92E-03 | -3,89E+00 |
| YALI1_C30749g | MFS domain-containing protein | 3,42E-07 | 1,17E-05 | -1,37E+00 |
| YALI1_C31684g | Vps8 domain-containing protein | 1,50E-02 | 4,64E-02 | 1,03E+00 |
| YALI1_C32886g | Acyl-coenzyme A oxidase | 1,15E-02 | 3,82E-02 | 3,73E+00 |
| YALI1_D00848g | E3 ubiquitin-protein ligase (EC 2.3.2.27) | 1,43E-03 | 7,89E-03 | -1,35E+00 |
| YALI1_D00951g | Peroxisomal membrane protein PEX17 | 3,39E-03 | 1,51E-02 | 1,77E+00 |
| YALI1_D01548g | Fungal-specific transcription factor domain-domain-containing protein | 9,42E-12 | 2,90E-09 | 4,08E+00 |
| YALI1_D01632g | Zn(2)-C6 fungal-type domain-containing protein | 1,21E-02 | 3,95E-02 | -1,97E+00 |
| YALI1_D01780g | tRNA wybutosine-synthesizing protein 2 (tRNA-yW-synthesizing protein 2) (tRNA(Phe) (4-demethylwyosine(37)-C(7)) aminocarboxypropyltransferase) | 1,59E-03 | 8,45E-03 | 2,53E+00 |
| YALI1_D02063g | NRDE protein-domain-containing protein | 9,79E-03 | 3,41E-02 | 1,36E+00 |
| YALI1_D02180g | N-glycosylation protein-domain-containing protein | 1,21E-03 | 6,89E-03 | -2,01E+00 |
| YALI1_D02348g | U1 small nuclear ribonucleoprotein component SNU71 | 2,32E-05 | 2,99E-04 | -1,77E+00 |
| YALI1_D02990g | Fungal-specific transcription factor domain-domain-containing protein | 5,08E-06 | 9,48E-05 | 2,11E+00 |
| YALI1_D03061g | HAD-like domain-containing protein | 2,33E-03 | 1,13E-02 | 1,15E+00 |
| YALI1_D03167g | ATP-dependent DNA helicase (EC 3.6.4.12) | 5,42E-03 | 2,15E-02 | -1,76E+00 |
| YALI1_D03805g | Peptidase S8/S53 domain-containing protein | 3,36E-05 | 3,94E-04 | -1,30E+00 |
| YALI1_D04469g | USP domain-containing protein | 3,07E-05 | 3,72E-04 | 1,31E+00 |
| YALI1_D04978g | ICE2-domain-containing protein | 1,58E-02 | 4,81E-02 | 1,56E+00 |
| YALI1_D06700g | Methionine aminopeptidase 2 (MAP 2) (MetAP 2) (EC 3.4.11.18) (Peptidase M) | 4,95E-04 | 3,46E-03 | 2,23E+00 |
| YALI1_D07118g | tRNA (guanine(37)-N1)-methyltransferase (EC 2.1.1.228) (M1G-methyltransferase) (tRNA [GM37] methyltransferase) (tRNA methyltransferase 5) | 1,28E-02 | 4,12E-02 | -9,40E-01 |
| YALI1_D07271g | VID27 cytoplasmic protein-domain-containing protein | 5,88E-05 | 6,05E-04 | 1,50E+00 |
| YALI1_D08031g | Ubiquitin-activating enzyme E1-like | 3,85E-04 | 2,80E-03 | 9,63E-01 |
| YALI1_D08564g | Iron permease FTR1/Fip1/EfeU | 6,62E-05 | 6,68E-04 | -7,56E-01 |
| YALI1_D09103g | Ras family-domain-containing protein | 3,60E-03 | 1,57E-02 | -1,25E+00 |
| YALI1_D09277g | Cupredoxin | 1,00E-02 | 3,47E-02 | 1,02E+00 |
| YALI1_D09766g | Fe2OG dioxygenase domain-containing protein | 1,22E-05 | 1,86E-04 | -2,63E+00 |
| YALI1_D09964g | Dynein light chain | 5,88E-03 | 2,27E-02 | -1,50E+00 |
| YALI1_D10565g | GTP cyclohydrolase N terminal-domain-containing protein | 1,25E-06 | 3,21E-05 | 1,94E+00 |
| YALI1_D10768g | Major facilitator superfamily domain-containing protein | 8,89E-04 | 5,46E-03 | -2,20E+00 |
| YALI1_D10858g | 37S ribosomal protein S24, mitochondrial | 3,74E-05 | 4,31E-04 | -1,58E+00 |
| YALI1_D11666g | HSM3_N domain-containing protein | 3,82E-03 | 1,63E-02 | 2,42E+00 |
| YALI1_D11686g | Histidine phosphatase superfamily | 3,62E-04 | 2,68E-03 | 1,90E+00 |
| YALI1_D11722g | AdoMet-dependent rRNA methyltransferase SPB1 (EC 2.1.1.-) (2'-O-ribose RNA methyltransferase) (S-adenosyl-L-methionine-dependent methyltransferase) | 1,05E-03 | 6,13E-03 | -1,24E+00 |
| YALI1_D12543g | SWR1-complex protein 4 | 4,09E-04 | 2,92E-03 | -1,24E+00 |
| YALI1_D12626g | U1 small nuclear ribonucleoprotein component SNU71 | 9,87E-03 | 3,43E-02 | 1,43E+00 |
| YALI1_D12880g | UBX domain-containing protein | 8,12E-04 | 5,14E-03 | -3,53E+00 |
| YALI1_D13341g | Zn(2)-C6 fungal-type domain-containing protein | 7,75E-03 | 2,87E-02 | -6,11E-01 |
| YALI1_D13545g | Aspartic peptidase domain-containing protein | 1,20E-02 | 3,92E-02 | 9,44E-01 |
| YALI1_D13790g | Ribosomal protein S5 domain 2-type protein | 3,58E-04 | 2,66E-03 | 2,35E+00 |
| YALI1_D13916g | SH3 domain-containing protein | 5,61E-03 | 2,20E-02 | 6,00E-01 |
| YALI1_D14224g | DBR1 domain-containing protein | 1,41E-02 | 4,45E-02 | 9,49E-01 |
| YALI1_D16086g | Nuclear fusion protein KAR5 (Karyogamy protein 5) | 1,16E-02 | 3,83E-02 | -7,65E-01 |
| YALI1_D16173g | Ubiquitin thioesterase OTU1 (EC 3.4.19.12) | 7,27E-04 | 4,63E-03 | 2,28E+00 |
| YALI1_D16249g | Ribosomal_L16 domain-containing protein | 1,24E-03 | 7,01E-03 | 1,19E+00 |
| YALI1_D17564g | Enhancer of mRNA-decapping protein 3 | 1,01E-02 | 3,48E-02 | 4,11E-01 |
| YALI1_D18037g | Acyl carrier protein | 3,39E-03 | 1,51E-02 | -1,23E+00 |
| YALI1_D18130g | Aspartate/ornithine carbamoyltransferase | 3,74E-03 | 1,62E-02 | 1,91E+00 |
| YALI1_D18943g | HECT domain-containing protein | 3,82E-03 | 1,63E-02 | 1,44E+00 |
| YALI1_D19951g | Amino acid permease-domain-containing protein | 1,42E-02 | 4,47E-02 | -5,60E-01 |
| YALI1_D20329g | DUF2470 domain-containing protein | 1,45E-02 | 4,55E-02 | 7,83E-01 |
| YALI1_D20473g | Phenylalanine--tRNA ligase (EC 6.1.1.20) | 1,54E-02 | 4,71E-02 | 1,70E+00 |
| YALI1_D20651g | Deacetylase sirtuin-type domain-containing protein | 5,21E-06 | 9,60E-05 | -1,67E+00 |
| YALI1_D20886g | Serine hydrolase FSH | 3,59E-03 | 1,57E-02 | -1,90E+00 |
| YALI1_D21023g | Cytidine deaminase (EC 3.5.4.5) (Cytidine aminohydrolase) | 5,03E-05 | 5,35E-04 | 6,53E-01 |
| YALI1_D22053g | DUF4451 domain-containing protein | 6,60E-03 | 2,51E-02 | -9,88E-01 |
| YALI1_D22247g | Signal recognition particle receptor subunit beta | 1,27E-03 | 7,16E-03 | -1,28E+00 |
| YALI1_D22274g | MFS domain-containing protein | 7,36E-05 | 7,30E-04 | 1,56E+00 |
| YALI1_D22401g | F-box domain-containing protein | 1,54E-02 | 4,73E-02 | -2,40E+00 |
| YALI1_D22768g | tRNA dimethylallyltransferase (EC 2.5.1.75) | 1,25E-02 | 4,07E-02 | -1,13E+00 |
| YALI1_D22997g | Fn3_like domain-containing protein | 8,07E-05 | 7,93E-04 | 1,67E+00 |
| YALI1_D24333g | Triacylglycerol lipase (EC 3.1.1.3) | 8,73E-06 | 1,42E-04 | -1,12E+00 |
| YALI1_D24375g | BRCT domain-containing protein | 1,48E-02 | 4,60E-02 | -1,41E+00 |
| YALI1_D25104g | IPPc domain-containing protein | 1,95E-03 | 9,86E-03 | -1,13E+00 |
| YALI1_D25359g | MFS domain-containing protein | 5,74E-05 | 5,94E-04 | -1,87E+00 |
| YALI1_D25968g | Zn(2)-C6 fungal-type domain-containing protein | 2,92E-04 | 2,26E-03 | 2,86E+00 |
| YALI1_D26186g | Mediator of RNA polymerase II transcription subunit 5 (Mediator complex subunit 5) | 4,02E-06 | 7,93E-05 | 2,97E+00 |
| YALI1_D26467g | P-loop containing nucleoside triphosphate hydrolase protein | 2,47E-04 | 1,96E-03 | -3,87E+00 |
| YALI1_D27148g | Inosine-5'-monophosphate dehydrogenase (IMP dehydrogenase) (IMPD) (IMPDH) (EC 1.1.1.205) | 4,43E-04 | 3,12E-03 | -1,19E+00 |
| YALI1_D27808g | MFS domain-containing protein | 5,86E-06 | 1,05E-04 | 1,69E+00 |
| YALI1_D27884g | Mitochondrial 54S ribosomal protein YmL19 | 1,49E-03 | 8,07E-03 | -3,27E+00 |
| YALI1_D27995g | Enhancer of translation termination 1 | 5,93E-09 | 4,68E-07 | 2,62E+00 |
| YALI1_D29109g | HMG box domain-containing protein | 1,66E-04 | 1,44E-03 | -1,13E+00 |
| YALI1_D29237g | DUF2433 domain-containing protein | 1,46E-03 | 7,97E-03 | 2,69E+00 |
| YALI1_D30285g | Triacylglycerol lipase (EC 3.1.1.3) | 3,52E-03 | 1,55E-02 | 4,46E-01 |
| YALI1_D30474g | SHNi-TPR domain-containing protein | 8,76E-03 | 3,15E-02 | -1,88E+00 |
| YALI1_D32070g | Mitochondrial import inner membrane translocase subunit TIM54 | 1,55E-05 | 2,20E-04 | -9,08E-01 |
| YALI1_D32619g | MFS domain-containing protein | 9,15E-04 | 5,51E-03 | 9,68E-01 |
| YALI1_D32880g | J domain-containing protein | 6,06E-03 | 2,34E-02 | -1,25E+00 |
| YALI1_D33380g | Cys/Met metabolism PLP-dependent enzyme-domain-containing protein | 5,36E-03 | 2,14E-02 | -2,29E+00 |
| YALI1_D34487g | DNA topoisomerase I (EC 5.6.2.1) (DNA topoisomerase 1) | 2,15E-06 | 4,87E-05 | 1,62E+00 |
| YALI1_D35398g | General transcription and DNA repair factor IIH | 1,27E-02 | 4,09E-02 | 1,42E+00 |
| YALI1_E00412g | Peptidylprolyl isomerase (EC 5.2.1.8) | 5,10E-03 | 2,06E-02 | 7,44E-01 |
| YALI1_E00510g | P-loop containing nucleoside triphosphate hydrolase protein | 5,49E-06 | 1,00E-04 | -2,73E+00 |
| YALI1_E00588g | Aldehyde dehydrogenase domain-containing protein | 4,83E-07 | 1,53E-05 | -1,03E+00 |
| YALI1_E00680g | LPG_synthase_C domain-containing protein | 2,22E-09 | 2,01E-07 | -1,42E+00 |
| YALI1_E00746g | Snf7-domain-containing protein | 2,76E-03 | 1,29E-02 | 1,08E+00 |
| YALI1_E00907g | Alpha/Beta hydrolase protein | 4,19E-05 | 4,64E-04 | 2,45E+00 |
| YALI1_E00928g | Ribosomal_L14e domain-containing protein | 2,00E-03 | 1,00E-02 | -4,76E-01 |
| YALI1_E01133g | ZIP zinc transporter-domain-containing protein | 7,49E-03 | 2,79E-02 | 9,46E-01 |
| YALI1_E01530g | Galactosyl transferase GMA12/MNN10 family-domain-containing protein | 1,56E-03 | 8,33E-03 | -6,83E-01 |
| YALI1_E01589g | C1-THFS protein | 3,34E-07 | 1,17E-05 | -1,29E+00 |
| YALI1_E02850g | Superoxide dismutase | 4,94E-03 | 2,01E-02 | 1,06E+00 |
| YALI1_E03150g | FHA domain-containing protein | 1,67E-04 | 1,44E-03 | -1,16E+00 |
| YALI1_E05119g | Threonyl/alanyl tRNA synthetase | 1,31E-02 | 4,19E-02 | -8,52E-01 |
| YALI1_E05446g | POT family-domain-containing protein | 9,04E-04 | 5,49E-03 | 1,03E+00 |
| YALI1_E05599g | UBC core domain-containing protein | 2,34E-05 | 3,00E-04 | -2,73E+00 |
| YALI1_E05866g | Anaphase-promoting complex subunit 2 | 1,52E-03 | 8,19E-03 | 1,23E+00 |
| YALI1_E06109g | VPS10 domain-containing protein | 1,12E-03 | 6,49E-03 | 1,29E+00 |
| YALI1_E06583g | SH3 domain-containing protein | 1,44E-03 | 7,92E-03 | 2,04E+00 |
| YALI1_E06843g | RNase H type-1 domain-containing protein | 6,53E-04 | 4,26E-03 | 1,65E+00 |
| YALI1_E07818g | Non-specific serine/threonine protein kinase (EC 2.7.11.1) | 1,10E-02 | 3,71E-02 | 8,18E-01 |
| YALI1_E08071g | Oligosaccharide translocation protein RFT1 | 3,02E-03 | 1,38E-02 | 1,20E+00 |
| YALI1_E08472g | Importin N-terminal domain-containing protein | 5,07E-03 | 2,05E-02 | -1,05E+00 |
| YALI1_E08689g | BTB domain-containing protein | 8,47E-05 | 8,22E-04 | 1,58E+00 |
| YALI1_E08709g | Thiamine pyrophosphate enzyme, N-terminal TPP binding domain-domain-containing protein | 2,27E-03 | 1,10E-02 | 1,29E+00 |
| YALI1_E09750g | Iron-sulfur clusters transporter ATM1, mitochondrial | 3,56E-03 | 1,57E-02 | -3,52E+00 |
| YALI1_E10013g | Hydrolase_4 domain-containing protein | 4,42E-05 | 4,81E-04 | -1,04E+00 |
| YALI1_E10210g | SRCR domain-containing protein | 1,46E-04 | 1,29E-03 | -1,11E+00 |
| YALI1_E10492g | V-type proton ATPase subunit H | 1,62E-02 | 4,90E-02 | 7,97E-01 |
| YALI1_E12711g | Transcription elongation factor 1 homolog | 3,95E-04 | 2,85E-03 | 2,20E+00 |
| YALI1_E12893g | Amino acid permease-domain-containing protein | 1,98E-05 | 2,62E-04 | -1,42E+00 |
| YALI1_E13287g | Ribosome biogenesis protein NSA2 homolog | 1,08E-02 | 3,66E-02 | 8,66E-01 |
| YALI1_E13329g | Major facilitator superfamily domain-containing protein | 1,44E-03 | 7,89E-03 | 1,32E+00 |
| YALI1_E13899g | Peroxisomal acetoacetyl-CoA thiolase | 8,10E-03 | 2,96E-02 | 9,64E-01 |
| YALI1_E14132g | HIT-type domain-containing protein | 6,43E-06 | 1,12E-04 | 1,76E+00 |
| YALI1_E14385g | Man1-Src1p-C-terminal domain-domain-containing protein | 9,32E-03 | 3,30E-02 | -1,00E+00 |
| YALI1_E15559g | 3-methylcrotonyl-CoA carboxylase 2 (EC 6.4.1.4) (3-methylcrotonyl-CoA:carbon dioxide ligase subunit beta) | 1,53E-04 | 1,35E-03 | 1,82E+00 |
| YALI1_E16174g | Nuclear protein localization protein 4 | 1,69E-03 | 8,86E-03 | 1,65E+00 |
| YALI1_E16325g | Cupin-like domain-domain-containing protein | 8,57E-04 | 5,36E-03 | -1,51E+00 |
| YALI1_E16493g | DnaJ domain-containing protein | 2,18E-05 | 2,83E-04 | 2,38E+00 |
| YALI1_E17583g | Spliceosomal protein DIB1 | 1,37E-03 | 7,66E-03 | 1,63E+00 |
| YALI1_E17754g | Anthranilate synthase (EC 4.1.3.27) | 9,58E-04 | 5,71E-03 | -2,46E+00 |
| YALI1_E17760g | Chromatin modification-related protein | 9,81E-04 | 5,80E-03 | 1,45E+00 |
| YALI1_E18802g | AP complex subunit beta | 2,99E-03 | 1,38E-02 | 1,41E+00 |
| YALI1_E18843g | Structural maintenance of chromosomes protein | 3,60E-03 | 1,57E-02 | -1,37E+00 |
| YALI1_E19201g | Protein-tyrosine-phosphatase (EC 3.1.3.48) | 1,32E-06 | 3,29E-05 | -1,44E+00 |
| YALI1_E19508g | Armadillo-type protein | 2,90E-03 | 1,34E-02 | 1,55E+00 |
| YALI1_E19757g | GATA-type domain-containing protein | 5,51E-03 | 2,17E-02 | -5,54E-01 |
| YALI1_E19916g | MULE domain-containing protein | 2,06E-05 | 2,70E-04 | -1,71E+00 |
| YALI1_E20070g | 2OG-FeII_Oxy domain-containing protein | 7,05E-10 | 7,75E-08 | -2,76E+00 |
| YALI1_E20269g | Protein transport protein SEC23 | 6,94E-04 | 4,47E-03 | -1,14E+00 |
| YALI1_E20361g | Phospholipid-transporting ATPase (EC 7.6.2.1) | 2,87E-05 | 3,54E-04 | 1,16E+00 |
| YALI1_E20766g | Pyridoxal phosphate-dependent transferase | 8,12E-03 | 2,96E-02 | -1,83E+00 |
| YALI1_E20995g | Haloacid dehalogenase-like hydrolase-domain-containing protein | 2,20E-04 | 1,80E-03 | -1,39E+00 |
| YALI1_E21832g | Myb-like domain-containing protein | 6,14E-03 | 2,36E-02 | -9,47E-01 |
| YALI1_E21915g | 4-aminobutyrate aminotransferase (EC 2.6.1.19) (GABA aminotransferase) (Gamma-amino-N-butyrate transaminase) | 3,16E-06 | 6,53E-05 | 1,89E+00 |
| YALI1_E22159g | Flavin_Reduct domain-containing protein | 8,10E-04 | 5,14E-03 | 1,41E+00 |
| YALI1_E22238g | Peroxisomal 3-oxoacyl-CoA thiolase | 2,88E-05 | 3,54E-04 | 2,70E+00 |
| YALI1_E22859g | Protein LOT5 | 1,33E-05 | 1,98E-04 | -2,38E+00 |
| YALI1_E23389g | SEC7 domain-containing protein | 5,50E-03 | 2,17E-02 | -6,34E-01 |
| YALI1_E23578g | Protein-serine/threonine kinase (EC 2.7.11.-) | 1,28E-04 | 1,17E-03 | 1,58E+00 |
| YALI1_E23809g | Chaperonin 10-like protein | 2,90E-06 | 6,29E-05 | 1,43E+00 |
| YALI1_E23988g | N-acetylglucosamine-6-phosphate deacetylase (EC 3.5.1.25) | 2,35E-04 | 1,89E-03 | 1,72E+00 |
| YALI1_E24245g | MFS domain-containing protein | 2,86E-03 | 1,32E-02 | -5,24E-01 |
| YALI1_E24513g | Putative RNA methyltransferase | 2,52E-03 | 1,20E-02 | 9,97E-01 |
| YALI1_E24735g | Peptidase A1 domain-containing protein | 1,19E-02 | 3,91E-02 | 7,35E-01 |
| YALI1_E26930g | Oxysterol-binding protein-domain-containing protein | 1,92E-03 | 9,73E-03 | 1,65E+00 |
| YALI1_E27406g | WD40-repeat-containing domain protein | 2,57E-06 | 5,77E-05 | 1,71E+00 |
| YALI1_E28046g | Shr3 amino acid permease chaperone | 1,76E-03 | 9,17E-03 | 1,11E+00 |
| YALI1_E28170g | WD40-repeat-containing domain protein | 2,10E-03 | 1,04E-02 | 1,13E+00 |
| YALI1_E28957g | Protein YIP | 1,37E-04 | 1,23E-03 | 3,12E+00 |
| YALI1_E31248g | DNA polymerase epsilon catalytic subunit (EC 2.7.7.7) | 1,96E-05 | 2,61E-04 | 2,41E+00 |
| YALI1_E31396g | Histone H2A | 1,48E-02 | 4,60E-02 | -2,70E+00 |
| YALI1_E32118g | Major facilitator superfamily domain-containing protein | 3,77E-03 | 1,62E-02 | -7,68E-01 |
| YALI1_E32241g | Major facilitator superfamily domain-containing protein | 1,01E-02 | 3,51E-02 | -6,86E-01 |
| YALI1_E32269g | DNA helicase (EC 3.6.4.12) | 1,44E-02 | 4,53E-02 | -2,52E+00 |
| YALI1_E32636g | Protein kinase domain-containing protein | 3,40E-03 | 1,51E-02 | 1,86E+00 |
| YALI1_E32816g | FK506-binding protein (EC 5.2.1.8) | 1,59E-03 | 8,43E-03 | 1,05E+00 |
| YALI1_E34525g | Vesicular-fusion protein SEC18 (EC 3.6.4.6) | 5,81E-04 | 3,90E-03 | -2,47E+00 |
| YALI1_E34586g | Homeobox domain-containing protein | 3,14E-04 | 2,40E-03 | -1,71E+00 |
| YALI1_E34621g | Nuclear cap-binding protein subunit 2 (20 kDa nuclear cap-binding protein) | 2,36E-05 | 3,02E-04 | -1,81E+00 |
| YALI1_E34708g | Sec1-like protein | 2,72E-05 | 3,39E-04 | -1,76E+00 |
| YALI1_E35780g | Mating factor alpha | 4,11E-03 | 1,73E-02 | -5,50E-01 |
| YALI1_E35988g | Protein transport protein SEC31 (Protein transport protein sec31) | 8,56E-03 | 3,10E-02 | 8,87E-01 |
| YALI1_E36437g | Acetyl-CoA deacylase (EC 3.1.2.1) (Acetyl-CoA hydrolase) | 9,60E-03 | 3,36E-02 | 4,99E-01 |
| YALI1_E36568g | Glycosyltransferase family 20-domain-containing protein | 5,03E-06 | 9,48E-05 | -1,51E+00 |
| YALI1_E37749g | DUF1992 domain-containing protein | 3,14E-03 | 1,43E-02 | 8,76E-01 |
| YALI1_E39057g | Dynein heavy chain, cytoplasmic (Dynein heavy chain, cytosolic) | 6,75E-03 | 2,55E-02 | -1,05E+00 |
| YALI1_E40282g | DUF676 domain-containing protein | 2,56E-03 | 1,22E-02 | -1,53E+00 |
| YALI1_E40324g | Mitochondrial ribosomal protein MRP51 | 6,33E-06 | 1,12E-04 | -1,55E+00 |
| YALI1_E40327g | Protein kinase domain-containing protein | 1,11E-03 | 6,42E-03 | 2,37E+00 |
| YALI1_E40671g | Catalase-like domain-containing protein | 1,15E-05 | 1,77E-04 | -1,68E+00 |
| YALI1_E41110g | RING-type domain-containing protein | 1,77E-03 | 9,17E-03 | 2,40E+00 |
| YALI1_E41251g | Catalase (EC 1.11.1.6) | 1,06E-02 | 3,60E-02 | -9,48E-01 |
| YALI1_E41325g | DDHD domain-containing protein | 7,70E-08 | 3,76E-06 | 1,66E+00 |
| YALI1_F00229g | AP-3 complex subunit delta | 5,74E-04 | 3,88E-03 | -1,05E+00 |
| YALI1_F02047g | SNF2 family N-terminal domain-domain-containing protein | 3,31E-03 | 1,48E-02 | 1,18E+00 |
| YALI1_F02697g | Wee1-like protein | 1,29E-03 | 7,23E-03 | 8,42E-01 |
| YALI1_F03529g | Sodium/hydrogen exchanger | 1,40E-02 | 4,42E-02 | -4,39E-01 |
| YALI1_F03842g | E2 ubiquitin-conjugating enzyme (EC 2.3.2.23) | 6,63E-08 | 3,35E-06 | 2,34E+00 |
| YALI1_F04314g | Kinase-like domain-containing protein | 4,75E-07 | 1,52E-05 | 2,16E+00 |
| YALI1_F05137g | Mediator of RNA polymerase II transcription subunit 15 | 1,15E-04 | 1,06E-03 | -1,06E+00 |
| YALI1_F06312g | NEDD8-activating enzyme E1 catalytic subunit (EC 6.2.1.64) | 2,55E-03 | 1,21E-02 | 8,25E-01 |
| YALI1_F06904g | Glycoside hydrolase | 1,15E-02 | 3,82E-02 | -2,40E+00 |
| YALI1_F07181g | DNA replication licensing factor MCM2 (EC 3.6.4.12) (Minichromosome maintenance protein 2) | 3,95E-03 | 1,68E-02 | -4,78E-01 |
| YALI1_F07793g | Acyl CoA binding protein-domain-containing protein | 2,83E-08 | 1,64E-06 | -2,20E+00 |
| YALI1_F08683g | AMPK1_CBM domain-containing protein | 5,87E-03 | 2,27E-02 | -6,47E-01 |
| YALI1_F09598g | Phosphoserine transaminase (EC 2.6.1.52) | 1,16E-03 | 6,67E-03 | 1,28E+00 |
| YALI1_F10122g | Major facilitator superfamily domain-containing protein | 9,65E-04 | 5,74E-03 | -1,00E+00 |
| YALI1_F10150g | PSP1 C-terminal domain-containing protein | 9,07E-08 | 4,26E-06 | -2,10E+00 |
| YALI1_F10184g | Extracellular mutant protein 11-domain-containing protein | 3,63E-03 | 1,58E-02 | -1,65E+00 |
| YALI1_F11292g | SGL domain-containing protein | 1,04E-03 | 6,12E-03 | 7,45E-01 |
| YALI1_F11481g | PKS_ER domain-containing protein | 6,84E-04 | 4,43E-03 | -6,12E-01 |
| YALI1_F11635g | NUDIX hydrolase domain-like protein | 1,21E-05 | 1,85E-04 | 1,38E+00 |
| YALI1_F11827g | Glutamate decarboxylase (EC 4.1.1.15) | 8,14E-05 | 7,98E-04 | 2,15E+00 |
| YALI1_F12033g | DNA-directed RNA polymerase III subunit RPC3 (RNA polymerase III subunit C3) | 2,02E-03 | 1,01E-02 | 1,68E+00 |
| YALI1_F12895g | Uricase (EC 1.7.3.3) (Urate oxidase) | 5,76E-03 | 2,24E-02 | -7,22E-01 |
| YALI1_F13046g | Atypical serine/threonine protein kinase BUD32 (EC 2.7.11.1) (Atypical serine/threonine protein kinase bud32) (EKC/KEOPS complex subunit BUD32) | 7,76E-03 | 2,87E-02 | -1,49E+00 |
| YALI1_F13170g | S-(hydroxymethyl)glutathione dehydrogenase (EC 1.1.1.284) | 5,38E-03 | 2,14E-02 | 7,97E-01 |
| YALI1_F13452g | ANL1P interacting protein | 2,24E-04 | 1,82E-03 | 1,62E+00 |
| YALI1_F14009g | Putative zinc finger in N-recognin-domain-containing protein | 1,56E-04 | 1,37E-03 | -1,14E+00 |
| YALI1_F14688g | CAAX prenyl protease (EC 3.4.24.84) | 1,52E-07 | 6,32E-06 | 2,13E+00 |
| YALI1_F14900g | G-patch domain-containing protein | 5,32E-06 | 9,75E-05 | 3,05E+00 |
| YALI1_F15291g | DUF4451 domain-containing protein | 2,61E-05 | 3,30E-04 | 8,67E-01 |
| YALI1_F15311g | Protein transport protein SFT2 | 2,71E-03 | 1,27E-02 | 9,24E-01 |
| YALI1_F15334g | DHS-like NAD/FAD-binding domain-containing protein | 3,20E-06 | 6,57E-05 | 1,93E+00 |
| YALI1_F15403g | GPI mannosyltransferase 2 (EC 2.4.1.-) | 9,16E-04 | 5,51E-03 | -4,80E-01 |
| YALI1_F15990g | X-domain of DnaJ-containing-domain-containing protein | 8,14E-04 | 5,15E-03 | -8,99E-01 |
| YALI1_F16045g | Endonuclease/exonuclease/phosphatase | 6,76E-04 | 4,39E-03 | 2,51E+00 |
| YALI1_F16117g | NMDA receptor-regulated protein 1-domain-containing protein | 1,18E-02 | 3,88E-02 | 6,77E-01 |
| YALI1_F16856g | Metallo-dependent phosphatase-like protein | 2,79E-03 | 1,30E-02 | -9,18E-01 |
| YALI1_F17337g | Ammonium transporter | 4,92E-07 | 1,54E-05 | -1,04E+00 |
| YALI1_F17383g | Exocyst complex subunit Sec15-like-domain-containing protein | 4,34E-03 | 1,80E-02 | 2,23E+00 |
| YALI1_F17637g | Dibasic-processing endoprotease | 1,80E-05 | 2,45E-04 | -1,17E+00 |
| YALI1_F17805g | Zn(2)-C6 fungal-type domain-containing protein | 5,59E-03 | 2,19E-02 | -1,83E+00 |
| YALI1_F18276g | DH domain-containing protein | 4,32E-05 | 4,74E-04 | 1,54E+00 |
| YALI1_F19058g | Maf-like protein | 4,73E-03 | 1,93E-02 | 1,57E+00 |
| YALI1_F19697g | Thioesterase-like superfamily-domain-containing protein | 1,58E-02 | 4,80E-02 | -1,72E+00 |
| YALI1_F21778g | Histone acetyltransferase (EC 2.3.1.48) | 1,11E-04 | 1,03E-03 | -1,77E+00 |
| YALI1_F21908g | Major facilitator superfamily domain-containing protein | 3,72E-07 | 1,23E-05 | -1,62E+00 |
| YALI1_F21989g | M20_dimer domain-containing protein | 5,48E-03 | 2,17E-02 | -1,14E+00 |
| YALI1_F22908g | Diphthine methyl ester synthase (EC 2.1.1.314) | 2,15E-03 | 1,06E-02 | -1,68E+00 |
| YALI1_F23191g | Zn(2)-C6 fungal-type domain-containing protein | 6,98E-04 | 4,48E-03 | -1,23E+00 |
| YALI1_F23214g | Major facilitator superfamily domain-containing protein | 3,12E-06 | 6,53E-05 | 1,95E+00 |
| YALI1_F23595g | Mediator of RNA polymerase II transcription subunit 16 (Mediator complex subunit 16) | 5,85E-06 | 1,05E-04 | -9,94E-01 |
| YALI1_F25066g | Alcohol acetyltransferase | 9,14E-04 | 5,51E-03 | 1,81E+00 |
| YALI1_F25254g | Eukaryotic translation initiation factor 3 subunit A (eIF3a) (Eukaryotic translation initiation factor 3 110 kDa subunit homolog) (eIF3 p110) (Translation initiation factor eIF3, p110 subunit homolog) | 5,18E-06 | 9,60E-05 | -1,09E+00 |
| YALI1_F25650g | Derlin | 2,69E-03 | 1,26E-02 | 5,05E-01 |
| YALI1_F26427g | AA_permease domain-containing protein | 2,73E-04 | 2,14E-03 | -2,33E+00 |
| YALI1_F27716g | RING-type domain-containing protein | 1,27E-02 | 4,10E-02 | -9,90E-01 |
| YALI1_F29765g | Peroxin-3 (Peroxisomal biogenesis factor 3) | 1,69E-03 | 8,86E-03 | 2,14E+00 |
| YALI1_F30641g | Signal sequence receptor alpha subunit | 1,46E-06 | 3,61E-05 | -2,30E+00 |
| YALI1_F31053g | Autophagy-related protein 17 | 6,59E-03 | 2,51E-02 | 1,32E+00 |
| YALI1_F32120g | Major facilitator superfamily | 9,32E-04 | 5,59E-03 | 1,65E+00 |
| YALI1_F32764g | MICOS complex subunit MIC60 (Mitofilin) | 9,10E-07 | 2,55E-05 | 2,31E+00 |
| YALI1_F32880g | Glutathione S-transferase | 3,92E-03 | 1,67E-02 | -1,60E+00 |
| YALI1_F33103g | Ribonuclease H-like domain-containing protein | 5,88E-03 | 2,27E-02 | -7,42E-01 |
| YALI1_F33246g | Glycolipid 2-alpha-mannosyltransferase-domain-containing protein | 1,95E-04 | 1,64E-03 | -2,93E+00 |
| YALI1_F34387g | Kinesin-like protein | 1,02E-02 | 3,51E-02 | 1,16E+00 |
| YALI1_F34521g | Ras family-domain-containing protein | 1,60E-02 | 4,85E-02 | -6,92E-01 |
| YALI1_F35907g | DNA polymerase II subunit 2 (DNA polymerase epsilon subunit B) | 6,32E-04 | 4,14E-03 | 1,74E+00 |
| YALI1_F37963g | KRR1 small subunit processome component (KRR-R motif-containing protein 1) | 5,91E-07 | 1,73E-05 | -8,49E-01 |
| YALI1_F37986g | Protein BTN | 4,30E-03 | 1,79E-02 | 1,94E+00 |
| YALI1_F39009g | S1-like domain-containing protein | 5,41E-03 | 2,15E-02 | 6,31E-01 |
| YALI1_F39527g | GTPase activating protein | 3,80E-04 | 2,78E-03 | -8,91E-01 |
| YALI1_F39620g | 2-methylisocitrate lyase, mitochondrial | 1,53E-11 | 4,10E-09 | 2,92E+00 |
